# Insulin-like peptide 3 expressed in the silkworm possesses intrinsic disulfide bonds and full biological activity

**DOI:** 10.1038/s41598-017-17707-1

**Published:** 2017-12-11

**Authors:** Takatsugu Miyazaki, Masaaki Ishizaki, Hideo Dohra, Sungjo Park, Andre Terzic, Tatsuya Kato, Tetsuya Kohsaka, Enoch Y. Park

**Affiliations:** 1 Laboratory of Biotechnology, Research Institute of Green Science and Technology, Shizuoka University, 836 Ohya, Suruga-ku, Shizuoka, 422-8529 Japan; 2 Laboratory of Biotechnology, Division of Applied Biological Chemistry, College of Agriculture, Shizuoka University, 836 Ohya, Suruga-ku, Shizuoka, 422-8529 Japan; 3 Instrumental Research Support Office, Research Institute of Green Science and Technology, Shizuoka University, Shizuoka, 422-8529 Japan; 4 Department of Cardiovascular Diseases and Center for Regenerative Medicine, Mayo Clinic, 200 First Street SW, Rochester, MN 55905 USA; 5 Laboratory of Animal Reproduction and Physiology, Division of Applied Biological Chemistry, College of Agriculture, Shizuoka University, Shizuoka, 422-8529 Japan

## Abstract

Insulin-like peptide 3 (INSL3) is a member of the relaxin/insulin superfamily and is expressed in testicular Leydig cells. Essential for fetal testis descent, INSL3 has been implicated in testicular and sperm function in adult males via interaction with relaxin/insulin-like family peptide receptor 2 (RXFP2). The INSL3 is typically prepared using chemical synthesis or overexpression in *Escherichia coli* followed by oxidative refolding and proteolysis. Here, we expressed and purified full-length porcine INSL3 (pINSL3) using a silkworm-based *Bombyx mori* nucleopolyhedrovirus bacmid expression system. Biophysical measurements and proteomic analysis revealed that this recombinant pINSL3 exhibited the correct conformation, with the three critical disulfide bonds observed in native pINSL3, although partial cleavage occurred. In cAMP stimulation assays using RXFP2-expressing HEK293 cells, the recombinant pINSL3 possessed full biological activity. This is the first report concerning the production of fully active pINSL3 without post-expression treatments and provides an efficient production platform for expressing relaxin/insulin superfamily peptides.

## Introduction

Insulin-like peptide 3 (INSL3), previously called relaxin-like factor, is a member of the relaxin/insulin superfamily. INSL3 was originally discovered in a boar testis cDNA library screen^1^ and is a major product of fetal and adult Leydig cells. INSL3 is constitutively expressed and secreted as either an A-B heterodimer^2^ or an A-B-C single-chain form (Supplementary Fig. 1)^3,4^ and initiates intracellular signaling by binding with high affinity to its specific receptor, relaxin family peptide receptor 2 (RXFP2; originally called leucine-rich G protein-coupled receptor 8 [LGR8])^5,6^. Although INSL3 is essential for fetal testis descent as revealed by mouse models targeting either *Insl3* ^7,8^ or *Rxfp2* ^9,10^, there is compelling but inconclusive evidence that INSL3 contributes to sperm production as a germ cell survival/anti-apoptotic factor in rats^11,12^ and boars^13,14^ and as a germ cell proliferation factor in zebrafish^15^. Additionally, INSL3 likely plays a role in sperm function based on the RXFP2 expression observed in rodent^16,17^, human^17^, boar^18^, goat, ram and bull^19^ spermatozoa.

Thus, interest in INSL3 for both research and clinical applications is growing rapidly. Because INSL3 isolation methods are very limited, with a yield of approximately 11 μg per 300 to 400 g of pig or goat testes^3,4^, the purification of biologically active recombinant INSL3 would be a major step towards producing an uninterrupted supply. INSL3 has been produced either by solid-phase synthesis of the separate A- and B-domains and subsequent chain recombination^20^ or by oxidative refolding and enzymatic processing of recombinant single peptides expressed in *E*. *coli* ^21^. The A- and B-domains are joined by three disulfide bonds that are required for INSL3 biological activity. For example, the conversion of three disulfide bonds into isopeptide bonds decreased binding affinity for the receptor^22^, and analogs lacking the intra-chain disulfide bond showed no activity^23^. To date, the challenge of disulfide bond formation in prokaryotic cells has precluded the preparation of biologically active INSL3 using a recombinant expression system without post-expression treatment.

To prepare mammalian proteins with the correct post-translational modifications, baculovirus expression systems are often used because the host insect cells can post-translationally modify proteins like mammalian cells. We developed a *Bombyx mori* nucleopolyhedrovirus (BmNPV) bacmid^24^, which is a hybrid shuttle vector for *E*. *coli* and *B*. *mori*, to facilitate heterologous protein production without baculovirus construction or amplification. The recombinant BmNPV bacmid DNA is directly injected into silkworm larvae and pupae for foreign gene expression^25,26^. Using this system, we have successfully produced cellular, mitochondrial, and membrane proteins exhibiting proper folding and post-translational modifications^27–30^.

Here, we expressed porcine INSL3 (pINSL3) in silkworm larvae using the BmNPV bacmid expression system and examined its structural properties and biological activity. Recombinant pINSL3 was successfully collected from larval hemolymph and displayed the proper disulfide linkages, and the RXFP2-stimulating activity was comparable to human INSL3 (hINSL3). This is the first report describing the heterologous production of INSL3 in an active form without applying post-expression chemical modifications. These results demonstrate that the silkworm-based BmNPV bacmid expression system is useful for expressing proteins with complex disulfide bonds such as relaxin/insulin family peptides.

## Results

### Expression and purification of recombinant pINSL3

**Figure 1:**
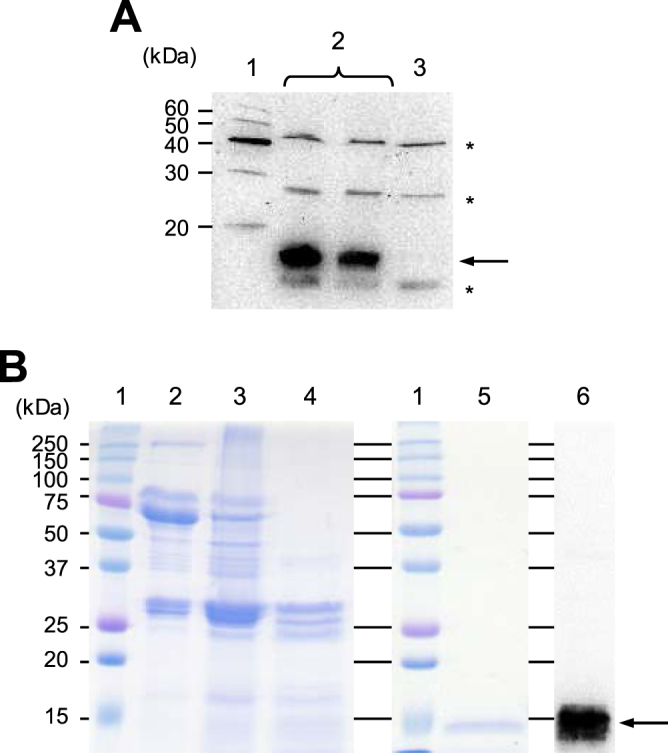
Expression and purification of recombinant pINSL3. (**A**) Western blot analysis of recombinant pINSL3 expressed in larvae hemolymph using an anti-DDDDK-tag antibody. *Lane 1*, molecular weight marker; *lane 2*, hemolymph from infected silkworms; *lane 3*, hemolymph from mock silkworms. The recombinant pINSL3 protein is indicated with an *arrow*, and non-specific silkworm proteins are *asterisks*. (**B**) SDS-PAGE analysis of the purification steps for recombinant pINSL3 detected by CBB staining (*lanes 1–5*) and western blotting (*lane 6*). *Lane 1*, molecular weight marker; *lane 2*, larva hemolymph; *lane 3*, supernatant after HCl treatment; *lane 4*, supernatant after neutralization; *lane 5*, affinity chromatography; *lane 6*, purified recombinant pINSL3 detected using an anti-FLAG-tag antibody.

FLAG-tagged pINSL3 was expressed in silkworm using the BmNPV bacmid system and successfully secreted into larval hemolymph using its native signal peptide (Fig. 1A). Because it was difficult homogenously purify the recombinant protein with traditional one-step affinity chromatography, the hemolymph was pre-treated with HCl to aggregate silkworm proteins. After acidification and neutralization, most of the silkworm proteins were eliminated (Fig. 1B, *lanes 3* and 4). Recombinant pINSL3 was further purified by FLAG-tag affinity chromatography, which showed a single 14 kDa band by SDS-PAGE and western blot analysis (Fig. 1B, *lanes 5* and 6) that was consistent with the theoretical molecular weight of 13,188 Da calculated from its amino acid sequence. The yield of purified recombinant pINSL3 was 8.3 μg from 10 mL of larval hemolymph.

### Secondary structure

**Figure 2:**
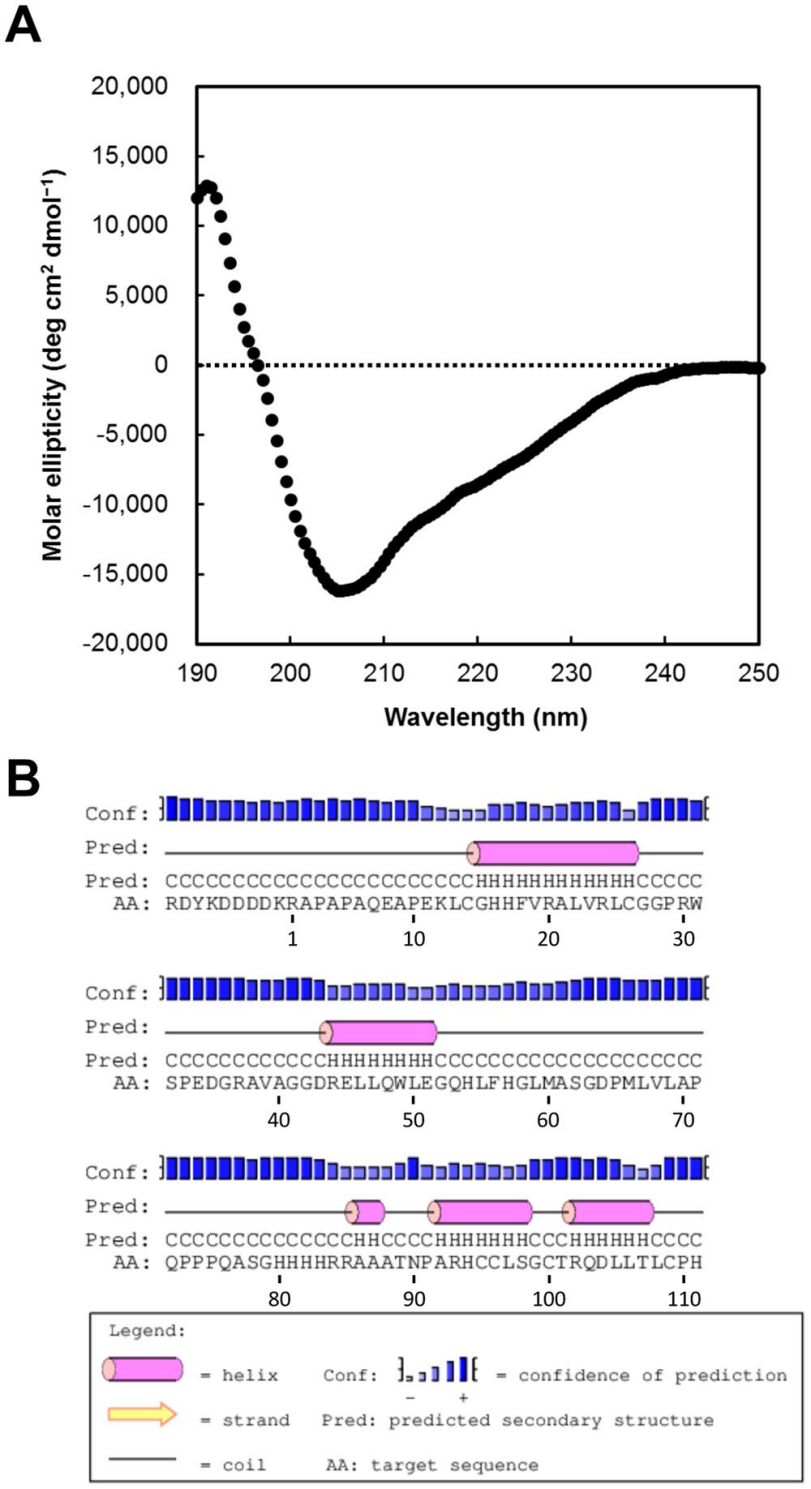
Secondary structure of recombinant pINSL3. (**A**) CD spectrum of recombinant pINSL3. (**B**) Secondary structure prediction for recombinant pINSL3 using the PSIPRED server v3.3.

Relaxin/insulin family peptides contain three α-helices that are functionally required^22,23^. The secondary structure of recombinant pINSL3 was analyzed by circular dichroism (CD) spectroscopy. The CD spectra show a maximum positive peak at 191 nm and a minimum negative peak at 205 nm (Fig. 2A), suggesting the presence of α-helices in the recombinant pINSL3 protein. The α-helix content was calculated to be 33%, which is similar to that of refolded recombinant hINSL3 expressed in *E*. *coli* (28%)^21^. The recombinant protein was estimated to contain five α-helices (Fig. 2B). One α-helix (Gly15–Cys26) should be located in the B-domain, and two moderate helices (Ala92–Ser98 and Arg102–Thr107) should be in the A-domain, which agrees with known structures of relaxin/insulin family peptides. Moreover, one α-helix (Arg44–Glu51) and a shorter α-helix (Ala86–Ala87) are predicted to be in the C-domain and N-terminus of the A-domain, respectively. The α-helix content based on the prediction is 29%, which is comparable to that calculated from the CD spectra.

### Structural analysis by LC-MS/MS

**Figure 3:**
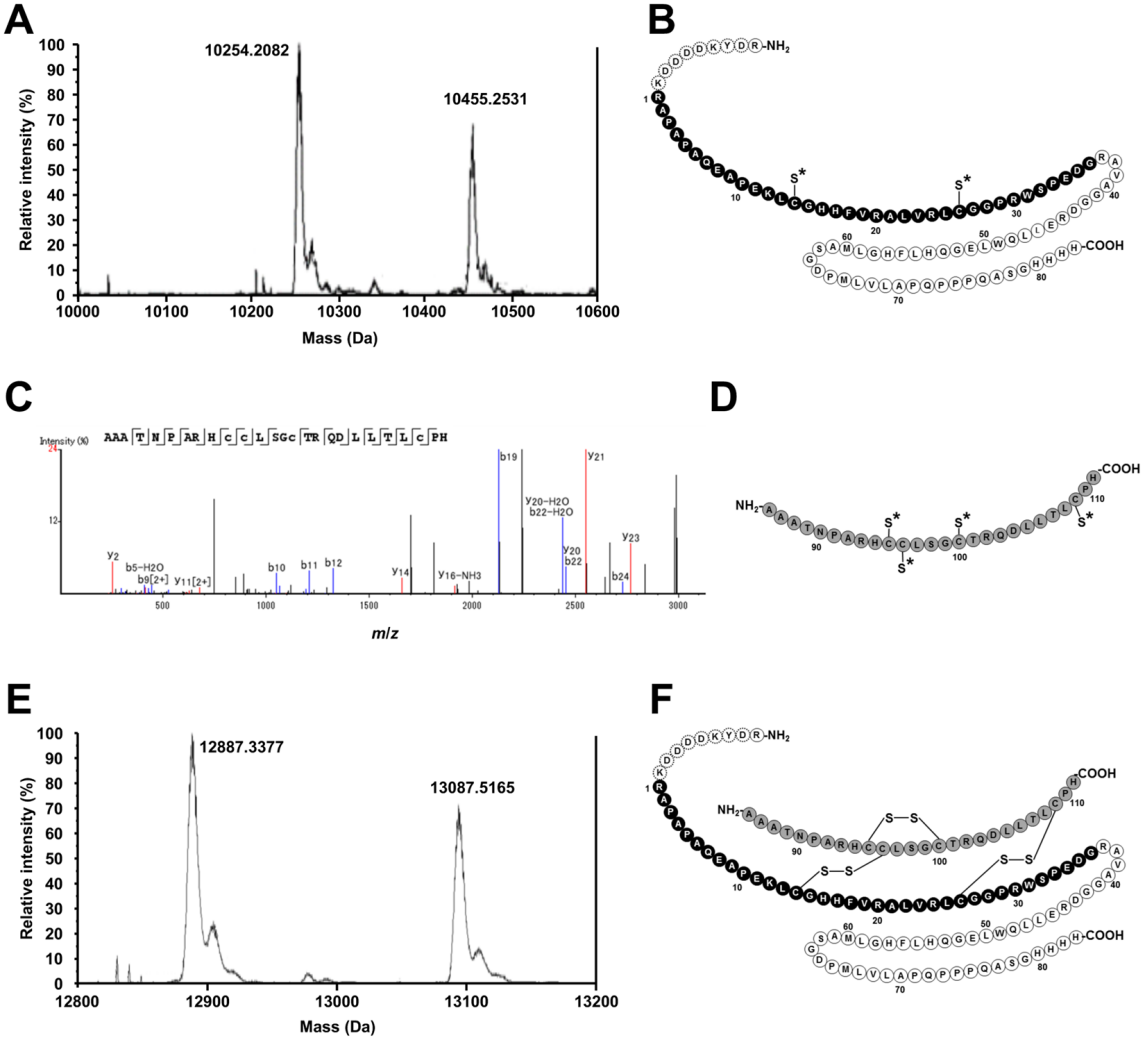
LC-ESI-TOF-MS spectra and schematic drawings of the predicted structure of recombinant pINSL3. The reduced and non-reduced recombinant pINSL3 samples were analyzed by LC-MS/MS to predict the structure of the recombinant pINSL3. (**A**) Deconvoluted mass spectrum for the reduced recombinant pINSL3 B-C chain. The reduced sample showed two peaks of 10254.2 Da and 10455.3 Da, which correspond to the FLAG-tagged B-C-chains and the same peptide with an N-terminally added Leu-Ser, respectively. (**B**) Structure of the B-C chain from reduced recombinant pINSL3 predicted by its molecular mass. (**C**) Product ion spectrum for the A-chain from reduced recombinant pINSL3. The reduced sample also showed a peak with m/z 745.86, and their product ions were well assigned to the amino acid sequence of the A-chain. (**D**) Structure of the A chain from reduced recombinant pINSL3 predicted by MS/MS product ion analysis generated by CID. (**E**) Deconvoluted mass spectrum for non-reduced recombinant pINSL3. Two peaks of 12887.3 Da and 13087.5 Da detected from the non-reduced sample correspond to the heterodimeric form of the FLAG-tagged B-C-domains and the A-domain, and the same form in which Leu-Ser is added to the N-terminus of the FLAG-tagged B-C-domains, respectively. (**F**) Structure of non-reduced recombinant pINSL3 predicted by the LC-MS/MS analysis results (**A–E**). The FLAG-tag, B-domain, C-domain, and A-domain residues are indicated by *dotted circles*, *black filled circles*, *unfilled circles*, and *gray filled circles*, respectively. Carbamidomethylated cysteine residue sulfhydryl groups are indicated with an *asterisk*.

To predict the structure of recombinant pINSL3, LC-MS/MS analysis was performed using reduced and non-reduced peptides (Fig. 3). The reduced sample showed two peaks with deconvoluted masses of 10254.2 Da and 10455.3 Da. The mass of 10254.2 Da was consistent with the theoretical mass of the FLAG-tagged peptide encoding the B- and C-domains (Arg-Asp-Tyr-Lys-Asp-Asp-Asp-Asp-Lys-Arg1–His83, 10254.4 Da) with cysteines carbamidomethylated by iodoacetamide for peptide mass fingerprinting (Fig. 3A,B). The latter peak corresponds to the theoretical mass of the same peptide with an N-terminally added Leu-Ser (10454.4 Da), which was probably generated by miscleavage from an endogenous signal peptidase. The LC-MS/MS analysis of the reduced sample also showed a peak with an *m*/*z* value of 745.86 ([M + 4 H]^4+^), which corresponds to the theoretical mass of the carbamidomethylated A-domain (Ala86–His111, 2979.37 Da) and was confirmed by MS/MS analysis (Fig. 3C,D). These results suggested that recombinant pINSL3 was hydrolyzed at two peptide bonds between His83 and Arg84 and between Arg85 and Ala86.

The non-reduced recombinant pINSL3 sample showed two major peaks with deconvoluted masses of 12887.3 Da and 13087.5 Da, both of which were lower than the theoretical mass of mature FLAG-tagged pINSL3 (Arg-Asp-Tyr-Lys-Asp-Asp-Asp-Asp-Lys-Arg1–His111, 13181.9 Da) (Fig. 3E). The 200 Da difference between these two peaks agreed with the additional mass of the N-terminal Leu-Ser residues described above. The mass of 12887.3 Da was consistent with the theoretical mass of a heterodimeric form (12887.5 Da) consisting of two polypeptides, including the FLAG-tagged B-C-domains (Arg-Asp-Tyr-Lys-Asp-Asp-Asp-Asp-Lys-Arg1–His83) and the A-domain (Ala86–His111), in which six cysteine residues are linked by disulfide bridges (Fig. 3F). These results suggest that recombinant pINSL3 protein comprises the B-, C-, and A-domains and contains three disulfide bonds like native pINSL3; the wild-type and recombinant protein only differ in terms of the internal cleavage between the C- and A-domains.

### HCD-MS/MS and ETD-MS/MS analysis of disulfide bond properties

**Figure 4:**
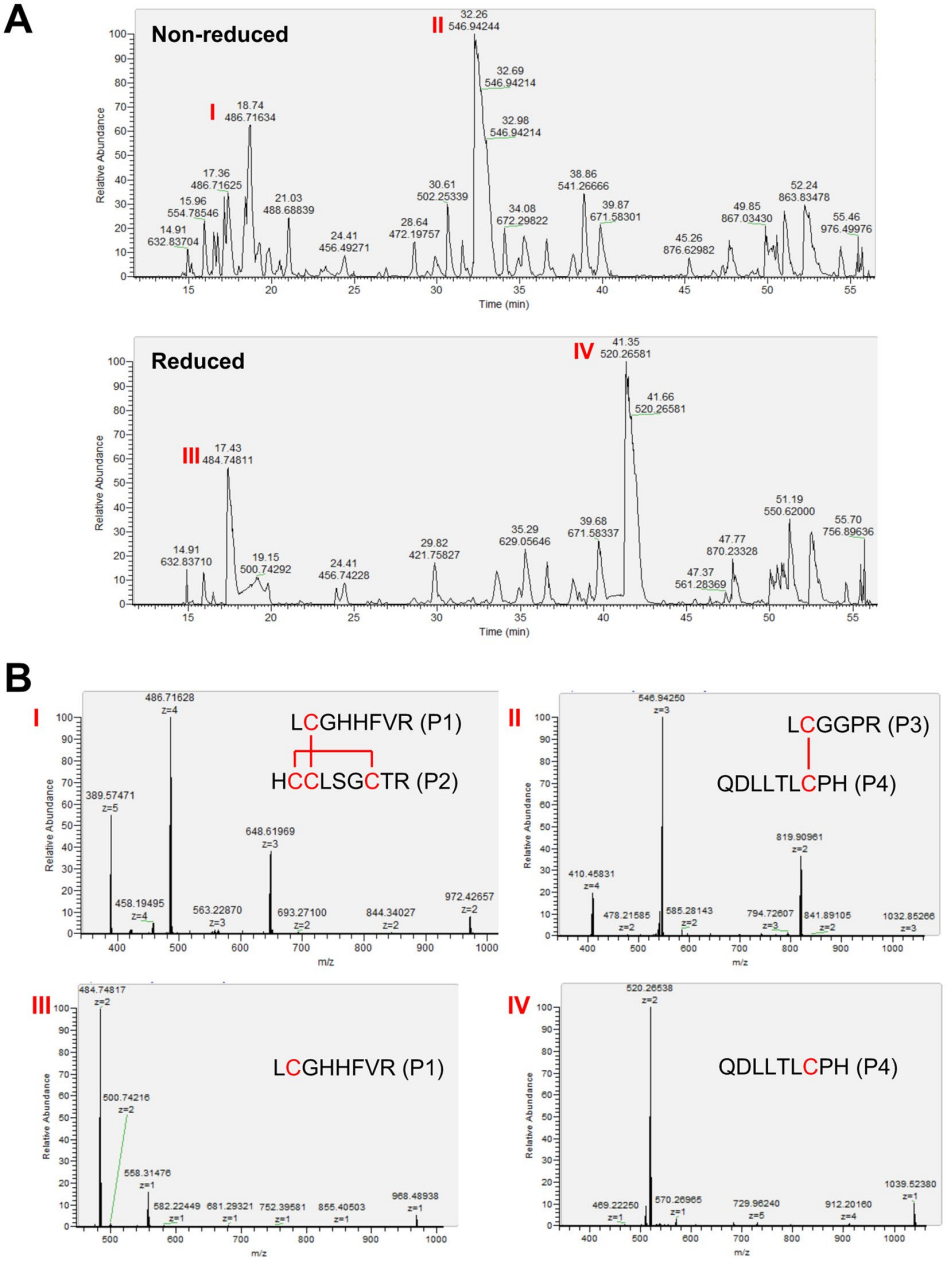
MS analysis of tryptic recombinant pINSL3. (**A**) Total ion chromatogram of tryptic-digested recombinant pINSL3 fragments displayed major peaks at 18.7 and 32.3 min (marked I and II) for non-reduced form and at 17.4 and 41.4 min (marked III and IV) for reduced form. (**B**) MS1 survey spectra of these marked peaks (I–IV) highlighted the cysteine peptide precursor ions. Based on accurate masses of the observed peptides, the peak I indicates that two peptides P1 (L^13^CGHHFVR^20^) and P2 (H^94^CCLSGCTR^102^) are connected by two disulfide bonds. The peak II implies that two peptides P3 (L^25^CGGPR^30^) and P4 (Q^103^DLLTLCPH^111^) are linked by a disulfide bond. Peaks III and IV suggest peptide P1 and P2, respectively.

To examine the disulfide bonds in recombinant pINSL3, the protein was digested and analyzed using higher-energy collisional dissociation (HCD) MS/MS and electron transfer dissociation (ETD) MS/MS. The total ion chromatograms of the digested peptides are shown in Fig. 4A. The two most intense peaks in the chromatogram (peaks I and II) were observed at 18.7 and 32.3 min, respectively, for the non-reduced peptides. The two major peaks (peaks III and IV) were also observed at 17.4 and 41.4 min, respectively, for the reduced peptides. The most abundant precursor ions in the survey spectra of these peaks are illustrated in Fig. 4B. The HCD-MS/MS analysis of the reduced digest identified cysteine-containing peptides P1 (residues 13–20, LCGHHFVR), P2 (94–102, HCCLSGCTR), P3 (25–30, LCGGPR), and P4 (103–111, QDLLTLCPH), which are summarized in Table 1. Peaks III and IV correspond to P3 and P4, which are in the B- and A-domains, respectively. Using the accurate masses of the observed HCD MS/MS matched peptides, peak I was predicted to be peptides P1 and P2, which are inter- and intramolecularly linked by two disulfide bonds; peak II was predicted to be peptides P3 and P4, which are intermolecularly linked by one disulfide bond.

**Table 1:** Identified cysteine peptides from recombinant pINSL3.

Non-reduced/reduced	*m*/*z*	Peptide fragments	Amino acid sequence	Percentage of total peak areas
Non-reduced	648.6 (3+)	P1 P2		25%
	546.9 (3+)	P3 P4		63%
	502.3 (4+) 669.3 (3+)	P1 P4		6%
	504.2 (4+) 672.3 (3+)	P2 P4		6%
Reduced	968.5	P1	LCGHHFVR	—
	977.4	P2	HCCLSGCTR	—
	602.3	P3	LCGGPR	—
	1039.5	P4	QDLLTLCPH	—

**Figure 5:**
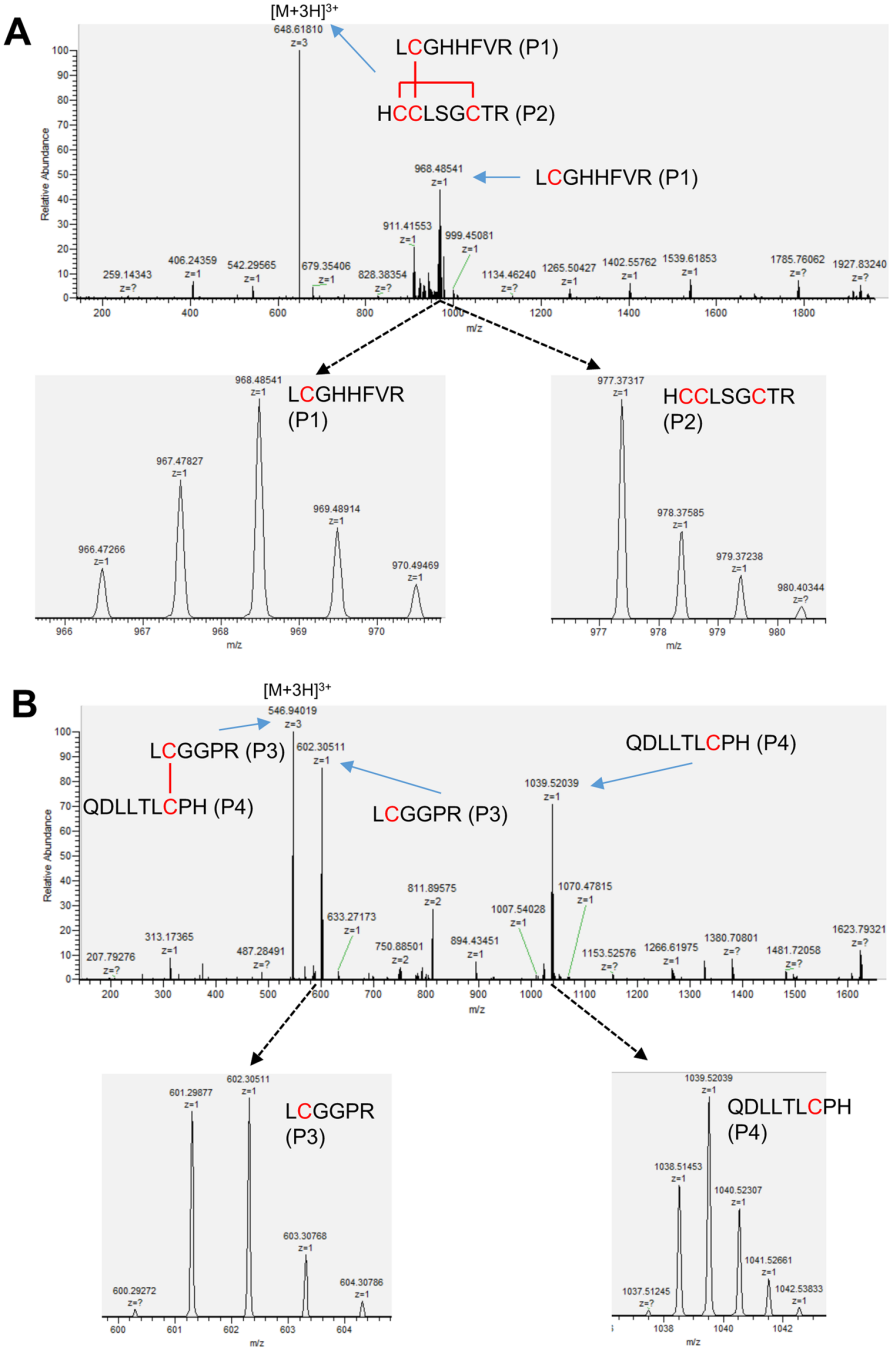
ETD-MS/MS analysis of the disulfide-linked peptides. (**A**) MS/MS spectra of the non-reduced ion with an *m*/*z* value of 648.6 (3+) from peak I in total ion chromatogram displayed characteristic peptide fragments P1 (L^13^CGHHFVR^20^) and P2 (H^94^CCLSGCTR^102^), indicating that Cys14 in P1 forms a disulfide bond with one of the cysteines in P2. (**B**) MS/MS spectra of the non-reduced ion with an *m*/*z* value of 546.9 (3+) from peak II in total ion chromatogram also generated distinctive peptide fragments P3 (L^25^CGGPR^30^) and P4 (Q^103^DLLTLCPH^111^), indicating that Cys26 in P3 forms an intermolecular disulfide bond with Cys109 in P4.

ETD fragmentation of the triply charged product ion with an *m*/*z* value of 648.62 (from peak I) showed two fragments, P1 and P2, suggesting that Cys14 in P1 forms a disulfide bond with Cys95, Cys96 or Cys100 in P2 (Fig. 5A; Supplementary Fig. 1). It would be challenging to identify which cysteine residues in P2 formed the intramolecular disulfide bond using this method. The ETD of the triply charged ion with an *m*/*z* value of 546.9 (from peak II) produced two fragments, P3 and P4, indicating that P3 and P4 formed an intermolecular disulfide bond between Cys26 and Cys109 (Fig. 5B). Unexpected disulfide pairs were also observed in *m*/*z* 502.3 [M + 3 H]^3+^ and 672.3 [M + 3 H]^3+^, each containing 6% of the total cysteine peptides detected (Table 1 and Supplementary Fig. 2). Collectively, these results suggest that most of the recombinant pINSL3 peptides possessed correct disulfide bond pairs.

### Biological activity

**Figure 6:**
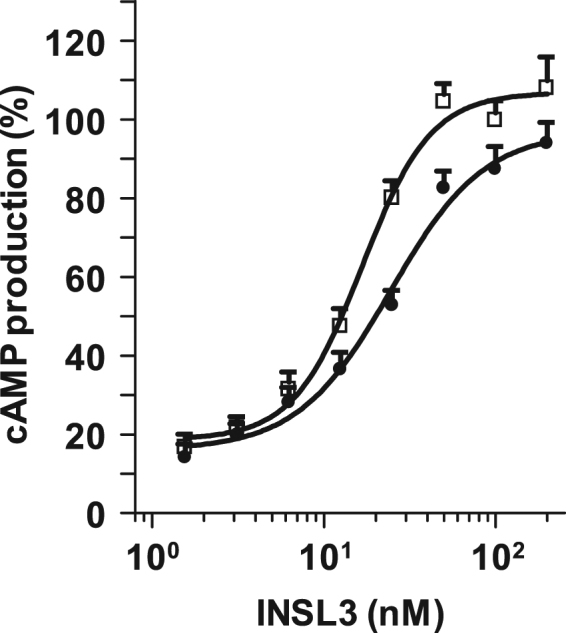
cAMP activity of recombinant pINSL3. Stimulation of cAMP production by recombinant pINSL3 (●) was compared to synthetic hINSL3 (□) in RXFP2-expressing HEK293 cells. cAMP production was expressed as a percentage of the maximum synthetic hINSL3 response to RXFP2. The values are expressed as the mean ± SEM of three independent experiments performed in triplicate.

The biological activity of the purified recombinant pINSL3 peptide was measured using a receptor-activating assay in RXFP2-expressing HEK293 cells. As shown in Fig. 6, cAMP production increased as the concentration of recombinant pINSL3 added to the reaction increased. Recombinant pINSL3 had an E_max_ (percentage of its maximum response) of 97.0 ± 5.6% (n = 12), which is comparable to that of the synthetic hINSL3 (106.8 ± 3.5%, n = 7). However, the EC_50_ of recombinant pINSL3 was 24.5 ± 1.2 nM (pEC_50_ = 7.61), which is slightly higher than that of standard hINSL3 consisting of the B- and A-domains (16.7 ± 1.1 nM, pEC_50_ = 7.78). These results suggest that the recombinant pINSL3 peptide was folded correctly and had ability to bind and stimulate its receptor to produce cAMP. However, the recombinant INSL3 is approximately 30% less active than synthetic INSL3 probably due to a decrease in affinity for the receptor as revealed by pEC_50_. The reason for the decline remains unknown, but it may be related to the presence of isomers.

## Discussion

In this study, full-length pINSL3 was successfully expressed in silkworm larvae using a BmNPV bacmid expression system and isolated using the following two steps: (1) acidic precipitation and (2) FLAG-tag affinity chromatography. To date, relaxin/insulin superfamily peptides including INSL3 have been prepared via complicated chemical synthesis or recombinant expression using *E*. *coli* followed by *in vitro* refolding^20,21,31,32^ because these proteins contain three evolutionarily conserved disulfide bonds. Although an *E*. *coli* expression system is easy to manipulate and yield a large amount of recombinant protein, many mammalian proteins are often expressed in insoluble inclusion bodies because the *E*. *coli* system is poor at posttranslationally modifying proteins^33^. Silkworms and other insect cells can posttranslationally modify recombinant proteins similar to mammalian cells, including glycosylation, phosphorylation and protein processing^29,34^. The silkworm-based BmNPV bacmid expression system produced fully active recombinant pINSL3 and might be an efficient tool for the preparation of other relaxin/insulin superfamily peptides.

The primary and secondary structures of the recombinant pINSL3 peptide were analyzed by LC-MS/MS and CD spectroscopy, respectively. Recombinant pINSL3 was found to mostly possess the correct fold, except for cleavage between the C- and A-domains. The C-domains of insulin and relaxin are processed by Golgi-localized endopeptidases, including prohormone convertase 1/3 (PC1/3)^35,36^ and furin^36–38^. However, native INSL3 extracted from boar testes is a single-chain peptide that likely possesses a C-domain^3^ because PC1/3 is not expressed in boar testes at any developmental stages^39^ and recombinant pINSL3 does not encode a cleavage motif (Arg-X-Lys/Arg-Arg) for furin in the C-peptide (-His-His-His-His-Arg-Arg-) C-terminus^4^. *B*. *mori* endogenously expresses the insulin-like peptide bombyxin, whose C-peptide is post-translationally processed by endopeptidases in a manner similar to the maturation of other relaxin/insulin family peptides^40,41^. Recombinant pINSL3 is likely hydrolyzed in the highly basic region of the C-domain by endopeptidases expressed in the Golgi apparatus of host silkworms in the secretion pathway. INSL3-stimulation of RXFP2 activity requires the α-helical structure of the B- and A-domains; native boar and goat INSL3 possess full bioactivity similar to synthetic hINSL3, which does not have a C-domain^3,4^. Although the relaxin/insulin superfamily C-domain has been reported to be required for proper precursor folding in the endoplasmic reticulum^42^, it is not fully understood whether the C-domain has any functions on its own. Although cleavage of the C-domain peptide in insulin is essential for its biological activity, it is evident that the INSL3 C-domain does not interfere with receptor binding, as its retention does not affect biological activity. Furthermore, the C-domain might contribute to the stability or half-life of INSL3 as in other peptide hormones^43,44^.

The ETD-MS/MS analysis suggested that recombinant pINSL3 mostly formed correct disulfide bonds, although the peptides derived from disulfide isomers were detected at 12% in total. Previous studies have demonstrated that recombinant insulin expressed in mammalian cells also includes non-native disulfide isomers^42,45–48^. The conserved disulfide bonds stabilize the α-helical structure of the B- and A-domains in INSL3 and are necessary for both binding affinity and activation of the RXFP2 receptor^22,23^. The disulfide isomers of other relaxin/insulin superfamily peptides are also inferior to native peptides with regards to bioactivity^49–52^. Despite the similarity of the E_max_ values between recombinant pINSL3 and synthetic hINSL3, the EC_50_ value of recombinant pINSL3 was slightly higher than that of the standard, suggesting that the disulfide isomers might act as partial agonists or antagonists of the receptor.

INSL3 has been characterized for its role in reproductive function such as testicular descent, germ cell survival and sperm fertility in males and the oocyte maturation and follicular development in females. Recently, it is also becoming important in extra-reproductive organs: in bones, INSL3 induces the cell proliferation and stimulate the differentiation of osteoblasts capable of mineralizing the extracellular matrix^53,54^. In eyes, INSL3 is expressed in the ocular surface and lacrimal gland, stimulates the proliferation of the conjunctival and sebaceous epithelial cell lines and accelerates corneal wound healing in corneal defect mice^55^. Additionally, INSL3 has been reported to be a powerful and multifunctional promoter of tumor growth and angiogenesis in human thyroid cancer cell xenografts^56^. It is evident, therefore, that INSL3 is not only physiologically but also clinically important. Application of recombinant INSL3 to reproductive, andrological, ophthalmic, bone and cancer fields helps to alleviate adverse symptoms in these areas. In the present study, successful production of fully active recombinant pINSL3 in silkworm larvae using a BmNPV bacmid expression system is a crucial step towards the production of recombinant human INSL3, thereby making the resulting products possible to be a convenient alternative to chemically synthesized human INSL3.

In summary, we successfully produced full-length pINSL3 using a silkworm-BmNPV bacmid expression system without oxidative refolding steps or additional post-expression modifications. The structural analyses revealed that the recombinant pINSL3 peptide adopted the correct conformation, with B- and A-domain α-helices and three crucial and evolutionally conserved disulfide bonds. The bioactivity assay demonstrated that the recombinant protein possessed full cAMP production capability. The silkworm expression system will facilitate the study of the structure-function relationship and clinical applications of INSL3. Furthermore, this study suggested that silkworm is an efficient host for the heterologous expression of functional proteins with complex disulfide bonds, including other relaxin/insulin superfamily peptides.

## Methods

### Recombinant pINSL3 BmNPV bacmid construction

To express recombinant pINSL3 as a secreted form in silkworm larva hemolymph and to facilitate its purification, a FLAG (DYKDDDDK) tag was inserted between its native signal sequence (20 amino acids) and the mature pINSL3 peptide. A DNA fragment (87 bp) encoding the secretion signal sequence and the FLAG-tag was amplified by PCR using primer 1 (forward) and primer 2 (reverse) and a pair of oligonucleotides, signal-FLAG-F and signal-FLAG-R (Table 2), as a template with the KOD-Plus-Neo DNA polymerase (Toyobo, Osaka, Japan). The pINSL3 B-C-A domain sequence (340 bp) was amplified by PCR using primer 3 (forward) and primer 4 (reverse) and the first-strand cDNA of pINSL3^3^ as a template. The mixture of these PCR products was used as a template for amplification of DNA encoding a precursor of FLAG-tagged-pINSL3 (sig-FLAG-pINSL3) using primer 1 and primer 4. The resulting DNA fragment was subcloned into the pFastBac1 vector (Thermo Fisher Scientific K. K., Yokohama, Japan) using an *Eco*RI restriction site. The construct was verified by DNA sequencing. *Escherichia coli* BmDH10Bac-*CP* ^−^-*Chi* ^−^ competent cells, which contains the cysteine protease- and chitinase-deficient BmNPV bacmid^26^, were transformed with the final plasmid and cultured on LB agar plate medium containing 50 μg/mL kanamycin, 7 μg/mL gentamycin, 10 μg/mL tetracycline, 40 μg/mL isopropyl β-D-1-thiogalactopyranoside, and 100 μg/mL 5-bromo-4-chloro-3-indolyl-4-galactoside (Takara Bio, Otsu, Japan) at 37 °C for 18 h. The recombinant BmNPV bacmid containing the sig-FLAG-pINSL3 gene was extracted from a white positive colony after blue-white selection.

**Table 2:** Primers used in this study.

Name	Sequence (5′ to 3′)
signal-FLAG-F	ATGGACCCCCACCCGCTCACCTGGGCTCTAGTGCTGCTGGGCCCGGCCCTGGCA
signal-FLAG-R	CTTGTCATCGTCATCCTTGTAGTCCCGGGAGAGTGCCAGGGCCGGGCCCAG
Primer 1	GAAGCGCGCGGAATTCATGGACCCCCACCCGCTC
Primer 2	CTTGTCATCGTCATCCTTG
Primer 3	AAGGATGACGATGACAAGCGGGCCCCTGCGCCCGCC
Primer 4	GTAGGCCTTTGAATTCAGTGGGGACAGAGGGTC

The EcoRI restriction sites are underlined.

### Expression and purification of recombinant pINSL3 from silkworm larvae

To produce recombinant pINSL3 protein in silkworm, 5 µg of the recombinant bacmid DNA was injected with 1,2-dimyristyloxypropyl-3-dimethyl-hydroxyethyl ammonium bromide (DMRIE-C, Thermo Fisher Scientific) directly into the dorsum of larvae using a syringe with a 26-gauge beveled needle (Terumo, Tokyo, Japan). The injected larvae were reared on an artificial diet (Silkmate 2 S, Nihon Nosan, Yokohama, Japan) at 26 °C for 6 days. The hemolymph was collected by cutting a caudal leg in a tube containing 1 mM 1-phenyl-2-thiourea, and it was stored at −80 °C until further analysis. The hemolymph was mixed with 3 volumes of 1 M HCl and centrifuged at 14,000 × *g* for 15 min. The supernatant was neutralized with 5 M NaOH. The insoluble material was removed by centrifugation at 14,000 × *g* for 15 min and clarified with a 0.45 μm filter. The sample was diluted with an equal volume of 20 mM Tris-HCl buffer (pH 7.5) containing 300 mM NaCl and 1% Triton X-100 and subjected to FLAG-tag affinity chromatography using a DDDDK-tagged Protein Purification Gel (Medical and Biological Laboratories, Nagoya, Japan). The column was washed with 20 mM Tris-HCl buffer (pH 7.5) containing 500 mM NaCl, and then the FLAG-tagged pINSL3 was eluted with 170 mM glycine-HCl buffer (pH 2.3) followed by neutralization with 1 M Tris-HCl (pH 8.0). The fractions containing the protein were concentrated in 20 mM Tris-HCl buffer (pH 8.0) or distilled water using an Amicon Ultra-15 3 K centrifugal filter unit (Millipore, Billerica, MA). Protein purification was performed at 4 °C. The protein concentration was determined using a Bio-Rad Protein Assay kit (Bio-Rad, Hercules, CA, USA) with bovine serum albumin as a standard.

### SDS-PAGE and Western blot analysis

The purity of recombinant pINSL3 protein was analyzed by tricine–SDS-PAGE (12% acrylamide gel) with Coomassie brilliant blue (CBB) staining and western blotting. The separated proteins on the gel were electroblotted onto a polyvinylidene fluoride membrane using the Mini Trans-Blot Electrophoretic Transfer Cell (Bio-Rad). The membrane was probed with an anti-DDDDK-tag monoclonal antibody (Medical and Biological Laboratories) as the primary antibody and an anti-mouse IgG antibody labeled with horseradish peroxidase (GE Healthcare, Buckinghamshire, UK) as the secondary antibody.

### Circular dichroism spectroscopy

The recombinant pINSL3 protein was dissolved in 20 mM Tris-HCl buffer (pH 8.0) and adjusted to 0.13 mg/mL for CD measurements, which were performed on a J-820 CD spectrometer (JASCO, Hachioji, Japan) at room temperature. The spectra were scanned from 250 to 195 nm in a cell with a 0.1 cm path length. The α-helix content was calculated from the mean residual ellipticity at 222 nm according to Chen and Yang^57^. Prediction of the secondary structure of recombinant pINSL3 was performed using the PSIPRED server v3.3^58^.

### Nano-LC-MS/MS analysis using CID fragmentation

Recombinant pINSL3 LC–MS/MS analysis was performed on a NanoFrontier eLD (Hitachi High-Technologies, Tokyo, Japan) linear ion trap time-of-flight mass spectrometer (LIT–TOF MS) coupled to a nanoflow NanoFrontier nLC HPLC (Hitachi High-Technologies Corporation). The recombinant pINSL3 was reduced with 25 mM dithiothreitol in 50 mM ammonium carbonate (pH 8.0) at 60 °C for 1 h followed by alkylation with 40 mM iodoacetamide. The reduced and non-reduced samples were dissolved in 0.3% formic acid, injected into the NanoFrontier nLC, trapped and desalted with a C18 monolith trap column (0.05 mm ID × 150 mm long; Hitachi High-Technologies Corporation), and then loaded onto a MonoCap C18 Fast-flow column (0.05 mm ID × 150 mm long; GL Sciences, Inc., Tokyo, Japan). Chromatography was performed using 0.1% formic acid in both solvent A (98% water/2% acetonitrile) and solvent B (98% acetonitrile/2% water), and the samples were eluted with a linear gradient from 5% B to 40% B over 110 minutes at 200 nL/min. The eluent was ionized with a nanoelectrospray ionization source equipped with an uncoated SilicaTip (New Objective, Woburn, MA, USA) and analyzed with a LIT–TOF MS. Mass spectra were obtained in positive ion mode at a scan mass range *m*/*z* from 200–2000. MS/MS spectra were generated by collision-induced dissociation (CID) in the linear ion trap. To calculate the mass of the recombinant pINSL3 and predict its structure, the mass spectra were deconvoluted using the NanoFrontier eLD Data Processing software (Hitachi High-Technologies Corporation). To confirm the amino acid sequence of the reduced recombinant pINSL3 A-chain, the MS/MS data were analyzed using PEAKS Studio version 7.0 (Bioinformatics Solutions, Inc., Waterloo, ON, Canada)^59^.

### Nano-LC-MS/MS analysis using HCD and ETD fragmentation

The recombinant pINSL3 protein was digested with 30 µL (0.005 µg/µL) trypsin (Promega, Madison, WI, USA) in 25 mM Tris (pH 8.1)/0.0002% Zwittergent 3-16 overnight at 37 °C, after which half of the volume was removed and reduced with 2 µL 0.5 M Tris(2-carboxyethyl)phosphine (TCEP) at 60 °C for 30 min. The non-reduced and reduced samples were acidified with 25 µL 2% trifluoroacetic acid and concentrated to less than 2 µL in a spinning vacuum concentrator before adding 0.15% formic acid/0.05% trifluoroacetic acid for protein analysis by nano-flow liquid chromatography electrospray tandem mass spectrometry (nanoLC-ESI-MS/MS) using a Thermo Scientific Orbitrap Elite Hybrid Mass Spectrometer (Thermo Fisher Scientific, Bremen, Germany) coupled to a Thermo Ultimate 3000 RSLC nano HPLC system. The digested peptide mixture was loaded onto a 250 nL OPTI-PAK trap (Optimize Technologies, Oregon City, OR, USA) that was custom-packed with Michrom Magic C18 solid phase (Michrom Bioresources, Auburn, CA, USA). Chromatography was performed using 0.2% formic acid in both solvent A (98% water/2% acetonitrile) and solvent B (80% acetonitrile/10% isopropanol/10% water) followed by a 5% to 45% B gradient over 45 minutes at 400 nL/min through a hand-packed PicoFrit (New Objective, Woburn, MA, USA) 100 mm × 350 cm column (Agilent Poroshell 120 EC-C18). The Orbitrap Elite mass spectrometer experiment performed a Fourier transform (FT) full scan from 340–1600 *m*/*z* with the resolution set at 120,000 (at 400 *m*/*z*), followed by orbitrap HCD-MS/MS scans on the top 12 ions. Dynamic exclusion was set to 1, and the selected ions were placed on an exclusion list for 30 seconds. The FTMS automatic gain control (AGC) target was set to 1e6, and the MSn target was set to 4e5 with max ion inject times of 250 ms and 100 ms, respectively. The ETD experiment was an FT full scan from 340–1600 *m*/*z* with the resolution set at 120,000 (at 400 *m*/*z*), followed by orbitrap ETD MS/MS scans on the top 12 ions with the following conditions: (1) ETD source settings at 50 µA for reagent ion source emission, (2) −70 V for electron energy, and (3) chemical ionization (CI) pressure of 17. The ion time and AGC targets were set to 50 and 1e5, respectively, and charge states of 2 and higher were allowed. HCD and ETD fragmentation for LC-MS/MS analysis were performed at the Mayo Proteomic Core Facility.

Base peak chromatograms of the non-reduced and reduced trypsin digest solutions were used to reveal ion peaks that were different between the two samples, and the expected tryptic masses were used to calculate the possible disulfide pairs and then assigned to the changing peaks. The ETD-MS/MS analysis was used to confirm the presumed disulfide paired ions by showing MS/MS product ions that were predominantly intact cysteine peptides involved in the pairs.

### INSL3 bioassay

The cAMP activity assay was performed with HEK293 cells transfected with mouse RXFP2 as described previously^3,14^. HEK293 cells were maintained in Dulbecco’s modified Eagle’s medium (DMEM; Sigma-Aldrich, St. Louis, MO, USA) supplemented with 10% (v/v) fetal bovine serum, a 1:100 dilution of penicillin/streptomycin solution (Sigma-Aldrich) and 2 mM L-glutamine in a humidified environment containing 5% CO_2_ at 37 °C. After the cells seeded in 96-well plates (TPP Techno Plastic Product, Trasadingen, Switzerland) reached 80% confluence, transient transfection of the expression construct or empty vector was performed using Lipofectamine 2000 in Opti-MEM serum-free media (Thermo Fisher Scientific K. K.). After 24 h of culture, the transfected cells were incubated for 1 h in DMEM containing 200 µm isobutylmethylxanthine with or without purified recombinant pINSL3 or synthetic hINSL3 peptide (Phoenix Pharmaceuticals, Belmont, CA). Intracellular cAMP was detected using the cAMP Biotrak ELISA kit (GE Healthcare) and expressed as a percentage of the maximum ligand response for RXFP2. All of the experiments were repeated at least three times using cells from independent transfections. The EC_50_ was calculated by employing the non-linear curve fitting functions in GraphPad PRISM version 5.0 software (GraphPad Software, San Diego, CA, USA).

## Electronic supplementary material

Supplementary Information
