# Supplementary material for: Insulin-like peptide 3 expressed in the silkworm possesses intrinsic disulfide bonds and full biological activity
Source: Sci Rep. 2017 Dec 11;7:17339. doi: 10.1038/s41598-017-17707-1 (PMC5725452; doi:10.1038/s41598-017-17707-1)
Supplement: Supplementary file 1 — Supplementary Information [file 41598_2017_17707_MOESM1_ESM.pdf]

Supplementary information

Insulin-like peptide 3 expressed in the silkworm possesses intrinsic disulfide bonds and full biological activity

Takatsugu Miyazaki[1,2], Masaaki Ishizaki[2], Hideo Dohra[3], Sungjo Park[4], Andre Terzic[4], Tatsuya Kato[1,2], Tetsuya Kohsaka[5] and Enoch Y. Park[1,2*]

[1] Laboratory of Biotechnology, Research Institute of Green Science and Technology, Shizuoka University, 836 Ohya, Suruga-ku, Shizuoka 422-8529, Japan. [2] Laboratory of Biotechnology, Division of Applied Biological Chemistry, College of Agriculture, Shizuoka University, 836 Ohya, Suruga-ku, Shizuoka 422-8529, Japan. [3] Instrumental Research Support Office, Research Institute of Green Science and Technology, Shizuoka University, Shizuoka 422-8529, Japan. [4] Department of Cardiovascular Diseases and Center for Regenerative Medicine, Mayo Clinic, 200 First Street SW, Rochester, MN 55905, USA. [5] Laboratory of Animal Reproduction and Physiology, Division of Applied Biological Chemistry, College of Agriculture, Shizuoka University, Shizuoka 422-8529, Japan. [*]Correspondence and requests for materials should be addressed to E.Y.P. (e-mail: park.enoch@shizuoka.ac.jp)

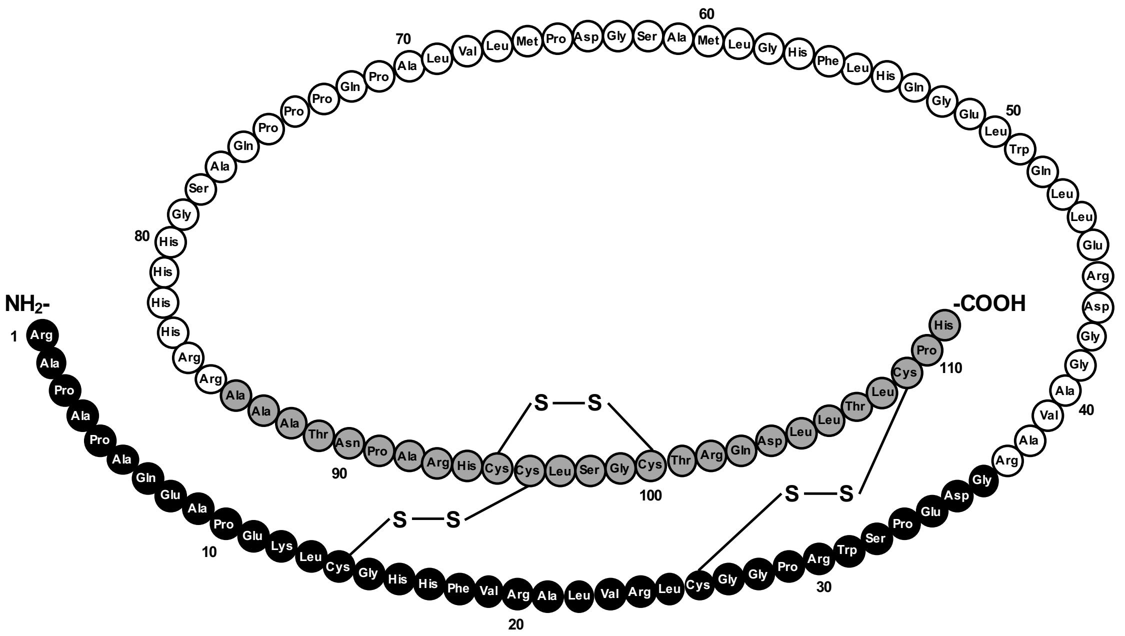

Supplementary Figure 1. Primary structure of native pINSL3. The B-domain, C-domain, and A-domain residues are indicated by *black filled circles*, *unfilled circles*, and *gray filled circles*, respectively.

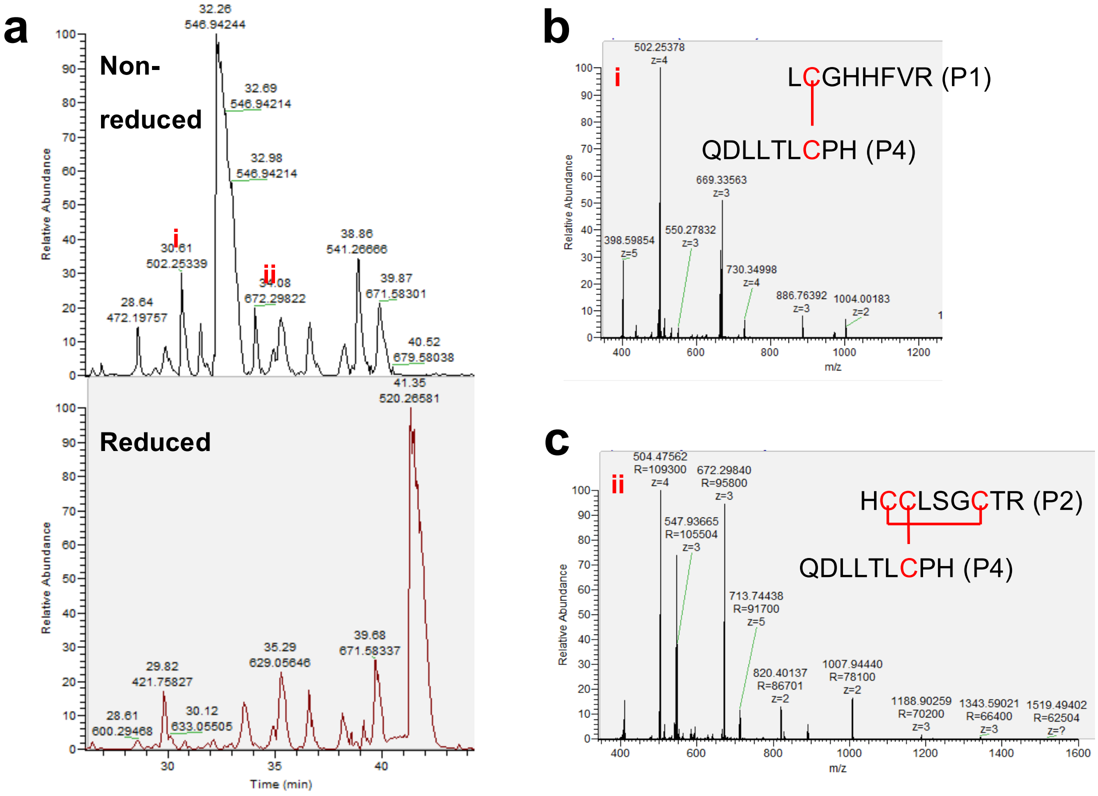

Supplementary Figure 2. MS analysis of unexpected disulfide-linked peptides in the recombinant pINSL3. (**a**) Total ion chromatogram of the non-reduced (upper) and reduced (lower) tryptic-digested recombinant pINSL3 fragments. Minor peaks from non-reduced form were eluted at 30.6 and 34.1 min, marked with i and ii, respectively. (**b and c**) MS1 survey spectra of the indicated peaks highlight the peptide precursor ions, which displayed unpredicted disulfide bond formation. Minor peaks i and ii suggest misfolded peptide fragments that Cys109 in P4 ($Q^{103}DLLTLCPH^{111}$) are connected with Cys14 in P1 ($L^{13}CGHHFVR^{20}$) or one of the cysteines in P2 ($H^{94}CCLSGCTR^{102}$), respectively.
